# The Effects of Parental Food Education on Children’s Food Literacy: The Mediating Role of Parent–Child Relationship and Learning Motivation

**DOI:** 10.3390/nu16152564

**Published:** 2024-08-04

**Authors:** Xing Xu, Huizi Cai, Jieying Zhang, Tiansheng Xia

**Affiliations:** School of Art and Design, Guangdong University of Technology, 729 Dongfeng E Rd., Guangzhou 510090, China

**Keywords:** food education, parent–child relationship, learning motivation, food literacy, teaching stage

## Abstract

Parental food education has been recognized among the important factors influencing children’s food literacy; however, the intrinsic mechanisms through which this influence occurs are unclear. In this study, a mediation model was constructed to explore this issue, using the parent–child relationship and learning motivation as mediating variables. In total, 204 children, aged 9–14 years old, responded to questionnaires on parental food education, children’s food literacy, the parent–child relationship, and learning motivation, which were used to measure the variables of interest. The results showed that parental food education was significantly and positively related to the parent–child relationship, learning motivation, and children’s food literacy; the parent–child relationship was significantly and positively related to learning motivation; and learning motivation was significantly and positively related to children’s food literacy. Parental food education influenced children’s food literacy in the following two main ways: the mediating role of learning motivation and the chain-mediating roles of the parent–child relationship and learning motivation. In addition, we attempt to explore the moderating role of the teaching stage between parental food education and the parent–child relationship, learning motivation, and children’s food literacy. In this paper, we discuss possible guidelines for family food education and children’s health based on the findings of the current study.

## 1. Introduction

Childhood overweight and obesity is becoming a major public health problem worldwide. The World Health Organization (WHO) reported that, by 2022, more than 390 million children and adolescents aged 5–19 would be overweight. The prevalence of overweight (including obesity) among children and adolescents aged 5–19 has risen dramatically, from 8% in 1990 to 20% by 2022 [1]. According to the latest data from the China National Nutritional Surveys (CNNSs), the prevalence levels of overweight and obesity among Chinese children and adolescents aged 6–17 years old are 11.1% and 7.9%, respectively, and China has ranked first in the world for the total number of obese children [2]. Overweight and obesity in childhood not only affect children’s physical and mental development [3], but also directly threaten their quality of life in adulthood [4]. Food literacy plays a key role in promoting healthier eating habits and overall well-being [5]; research has shown that children and adolescents who have higher levels of food literacy tend to make more nutritious food choices and adopt healthier and more sustainable eating habits, leading to greater health outcomes and a lower risk of diet-related illnesses, such as neophobia, overweight and obesity, and foodborne illnesses [6,7]. Most interventions aimed at improving children’s food literacy are focused on schools, but, although school-based interventions provide children with opportunities to engage in health-promoting activities, children’s food literacy remains unsatisfactory [8]. The family is the primary environment in which food choices and consumption patterns are established during childhood and later adolescence, and even though the eating environment becomes more complex and further from direct parental supervision when adolescents enter school [9], parents continue to have an important influence on adolescent food literacy. It is estimated that early adolescents burn up to 65% of their calories at home [10]. Parental health behaviors and practices have significant impacts on adolescent food literacy and can create a positive health-protective effect [11,12,13]; however, the relationship between the two, and the mechanisms of how parental food education affects children’s food literacy, are unclear, and research on this issue has important theoretical and practical value.

Over the past decade, more and more research has shown that parental factors play important roles in children’s food literacy, but less research has been conducted on the effects and mechanisms of interactions between parental food education methods and children’s food literacy and other relevant behaviors, especially in parental food education methods. Instead, previous research has focused on feeding practices such as controlling (coercive, pressuring, or restricting), condoning (allowing the child to have complete control over the feeding environment), non-participation practices, etc., with more emphasis on parental attitudes and beliefs about parenting in the feeding environment, in relation to teaching styles and specific teaching situations, but research on educational styles around feeding per se has appeared less in family scenarios and more in school scenarios. In addition, in the family scenario, because mothers often act as food providers and caregivers for the family, their role is often seen as the core focus of many food parenting studies that examine the impacts of parental behaviors on children’s diets, or the concept of the “parent” is equated with the “mother” [14,15,16], while the role of fathers is often overlooked and even considered a barrier to healthy eating [17]. Increased female employment and societal shifts in fathers’ child-rearing roles suggest that a mother-centered focus on dietary influences can impair a comprehensive understanding of family eating habits [18]. Moreover, a few studies have shown that father-responsive food education does affect children’s diets and diet-related health outcomes [19].

Children’s behavior is often influenced by the parent–child relationship, and diet is no exception, with research showing that children who grow up in positive parenting environments and family relationships have healthier levels of food literacy [20,21]; however, because existing research often views food interactions between parents and children as living behaviors, and rarely treats parental food education in the family as an education, there is less research on the mechanisms by which learning motivation is linked to parental food education styles and children’s food literacy. Researchers have found that parental teaching and participation can make children aware of parents’ importance and attitudes, thus improving children’s learning motivation, which can predict learning achievement or outcome [22]. It is worth noting that previous research has shown significant differences between teaching stages in terms of parental food education, parent–child relationships [23], learning motivation, and children’s food literacy [24,25]. However, there are no consistent findings as to whether the teaching stage makes a difference in the relationships among the four. Therefore, in this study, we examine the relationship between parental food education and children’s food literacy as a means of exploring the mediating roles of the parent–child relationship and learning motivation, as well as the moderating role of the teaching stage.

## 2. Literature Review and Hypotheses Development

### 2.1. Parental Food Education and Children’s Food Literacy

The term “food literacy” is a new concept, defined as, “the collection of interrelated knowledge, skills, and behaviors required for planning, managing, selecting, preparing, and consuming food to meet needs and determine nutritional intake [26]”. Food literacy, in addition to the focus on traditional nutrition education, requires an understanding of food shopping and preparation. Children who participate in food preparation at home are more likely to continue to enjoy cooking and preparing healthy meals as adults [26,27]. Moreover, studies have shown that the main food-related factors affecting childhood obesity are dietary quality and participation in food preparation [28]. In relevant food education programs, dietary quality generally includes vegetable and fruit intake, picky eating, wasteful behavior, and so on. Family dietary activities generally focus on helping family members cook, including washing dishes, arranging plates, preparing food, stir-frying, and so on [29].

Food education refers to the teaching of a wide range of food-related knowledge and skills to help people “eat right” [30]. In regard to the family scenario, this study defines parental food education, as parents teach children a wide range of food-related knowledge and skills to help children “eat right”. In existing studies, food education can be broadly categorized into theoretical and practical education, with theoretical education including videos, picture books, etc., and practical education including cooking, games, etc. [19,29,31].

Research has shown that parents cooking at home with their children reduces childhood obesity, and increases the frequency of children’s participation in dinner preparation and healthy food literacy [19]. In addition, a study showed that picture books have the potential to be a simple and convenient form of family nutrition education that can help parents try to reduce other poor eating habits, such as picky eating, in children. Not only can picture books be read slowly and used as many times as is convenient for the parent, but engaging children in discussions within the book can provide an opportunity for children to express their understanding of the story’s message and relate it to their personal behavior [32]. It is clear that parental food education increases children’s dietary quality and frequency of participation in family dietary activities. Therefore, we propose hypothesis H1:

**Hypothesis** **H1.** *Parental food education has a significant positive impact on children’s food literacy.*

### 2.2. The Mediating Effect of the Parent–Child Relationship

The parent–child relationship has been defined as a unique and influential relationship, established during parent–child interactions, which is critical to the physical and mental development of adolescents [33]. Food-related family activities are often recognized as having a strong correlation with the parent–child relationship [34], possibly because food is often closely linked to family health, so food-related family activities are more likely to trigger bonding among family members. In addition, food is central to the way children express love in their families [35], and food-related family interactions promote family connections, self-efficacy, and positive and healthy emotions [36]. Therefore, if parents conduct teaching activities around food, children will feel valued and cared for by their parents during these interactions, which can effectively improve parent–child relationship closeness.

Parent–child relationships usually impact children’s eating behaviors. One of the first studies on attachment phenomena in adolescents with eating disorders found that the presence of an affectively positive and emotionally supportive parental relationship, in conjunction with parental fostering of autonomy, reduced weight concerns, feelings of inadequacy, and overeating behaviors in young people [37], whereas poor relationships with parents can lead to a lack of family cohesion and obstacles to parent–child communication, which makes it less likely for children to participate in the family’s dietary activities [36]. It is evident that the quality of the parent–child relationship influences children’s food literacy. Therefore, we propose hypothesis H2:

**Hypothesis** **H2.** *The parent–child relationship mediates the relationship between parental food education and children’s food literacy.*

### 2.3. The Mediating Effect of Learning Motivation

Regarded as a necessary factor for students’ engagement in learning, motivation is defined as the motivation to perform or undertake a specific task [38]. Studies have shown that parental involvement increases children’s motivation and participation in learning activities by supporting and encouraging them to adopt a positive approach to achievement [39]. Existing food education activities organized by schools in conjunction with parents have been shown to increase children’s self-efficacy and interest in learning about food education, thereby improving children’s dietary quality and their frequency of participation in family dietary activities [29,40,41].

In addition, learning motivation is also recognized as one of the key factors influencing students’ engagement in learning, as learning motivation helps to establish high expectations, thereby encouraging children to actively participate in learning [42]. Therefore, if food education is viewed from a pedagogical perspective, an increase in children’s learning motivation promotes higher expectations and a sense of efficacy in food learning, which, in turn, affects their enjoyment, interest, and engagement in food learning [43], enabling them to demonstrate better learning behaviors and outcomes, and shaping better food literacy. Therefore, we propose hypothesis H3:

**Hypothesis** **H3.** *Learning motivation mediates the relationship between parental food education and children’s food literacy.*

Notably, when children interact with their parents, they can receive emotional support, and are more likely to feel respected and loved by their parents [44]. In general, the parent–child relationship contributes to the development of children’s learning motivation by reinforcing their need for relatedness, competence, and autonomy [45]. Thus, in a family food education scenario, parental food education can increase the interaction with children and build a more favorable parent–child relationship that enhances children’s learning motivation within the food education environment. Therefore, we propose hypothesis H4:

**Hypothesis** **H4.** *Parental food education influences children’s food literacy through the chain-mediated effects of the parent–child relationship and learning motivation.*

### 2.4. The Moderating Effect of the Teaching Stage

The teaching stage is a broader concept than grade level [46]. For the current study, grades 1–3 are generally referred to as the lower elementary grades; grades 4–6 are referred to as the upper elementary grades; grades 7–9 are referred to as the middle school grades; and grades 10–12 are referred to as the high school grades [47]. In order to more clearly examine the effects of different teaching stages on children’s diets, some studies categorize 9–10 year olds as elementary school students and 13–14 year olds as middle school students [24]. Because many children go through puberty around the same time, as they move from elementary school to middle school, with shifts in various aspects of cognition and behavior [48,49], the shift between teaching stages may also have an important impact on children’s food-related cognitions and behaviors.

First, as children transition from elementary school to middle school, they are required to spend more time and energy on attending school, participating in sports and extracurricular activities, and being with friends [24]. Second, upon entering middle school, parental food control begins to diminish, except for eating out, and children have more control over their food choices [24], which results in the gradual withdrawal of parents from the children’s eating circles. All of these factors can force parents, or unconsciously cause them, to reduce their children’s food education. As a result, parents reduce their food care for middle school students, so it is easier for middle school students to feel that their parents do not care for them, or even do not attach importance to them, than it is for elementary school students.

Moreover, as children gain greater independence with the transition to middle school, the parent–child relationship undergoes an important shift. Research shows that parent–child conflict increases and peaks in early adolescence. This increase in conflict is accompanied by a decrease in emotional intimacy, especially when children spend less time with their parents [50]. However, due to the new social and academic demands to which preadolescent children must adjust as they enter middle school, they may need more social support, and due to the disruption in their social networks after the transition, parents may be called upon to temporarily compensate for the loss of peer support at this time [23]. Therefore, when children are in middle school, food education, as an activity that can convey the level of parental care and attention, will exacerbate the deterioration in parent–child relationship quality even more if its frequency decreases. Therefore, we propose hypothesis H5:

**Hypothesis** **H5.** *The teaching stage plays a moderating role between parental food education and the parent–child relationship, and the moderating effect is greater at the middle school stage than at the elementary school stage.*

Although middle school students possess knowledge about healthy eating, their food preferences, especially in terms of taste, texture, and appearance, override this knowledge when making food decisions [51]. Additionally, middle school students tend to perceive parental feeding strategies and family diets as less important in influencing their food choices compared to elementary school students [24]. Elementary school students were more likely to be influenced by familial dietary patterns and parental advice compared to middle school students [52]. Thus, middle school students are prone to a lack of perceived utility value, interest, or expectation of food education provided by their parents, leading to difficulties in generating learning motivation for parental food education; middle school students are also more likely to be influenced by their peers, school, or their own preferences, so the situation in which parental food education is considered an education may decline at this time.

In addition, there is evidence that as children move from elementary to middle to high school, their consumption of breakfast, fruits, vegetables, and milk decreases, while their consumption of other beverages increases [53]. Eating out with peers or at school is one of the reasons middle school students may consume less healthy diets. At the same time, school has a heavier influence on middle school students’ diets than on those of elementary school students, and middle school students will consume unhealthier foods during the school day than at home [24], which also makes middle school students much less likely to participate in family dietary activities. These factors suggest that middle school students are more likely to have poorer food literacy and are less likely to be influenced by learning motivation than elementary school students. Therefore, we propose hypothesis H6:

**Hypothesis** **H6.** *The teaching stage plays a moderating role between motivation and children’s food literacy, with greater moderating effects at the elementary school stage than at the middle school stage.*

Through the above hypotheses, our purpose of the study is to examine the effects of parental food education on children’s food literacy.

## 3. Materials and Methods

### 3.1. Theoretical Model

**Figure 1:**
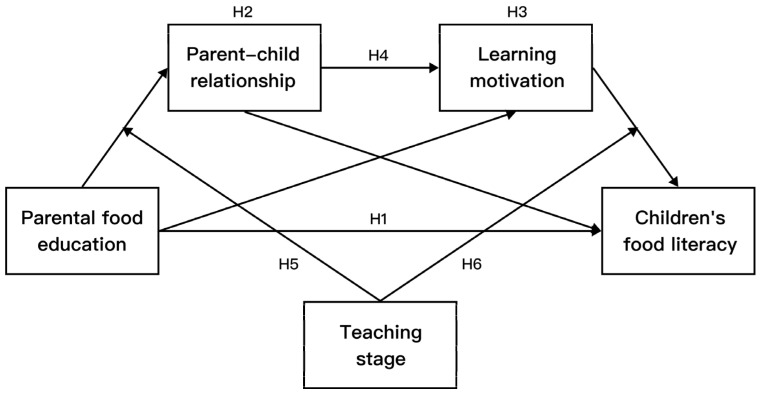
Theoretical model.

In summary, we constructed a model with moderated mediation (Figure 1) to systematically examine the effects of parental food education, the parent–child relationship, and learning motivation on children’s food literacy, as well as the moderating role of the teaching stage (elementary and middle school). Based on previous studies, we proposed the six following hypotheses: Hypothesis H1: Parental food education has a significant positive impact on children’s food literacy. Hypothesis H2: The parent–child relationship mediates the relationship between parental food education and children’s food literacy. Hypothesis H3: Learning motivation mediates the relationship between parental food education and children’s food literacy. Hypothesis H4: Parental food education influences children’s food literacy through the chain-mediated effects of the parent–child relationship and learning motivation. Hypothesis H5: The teaching stage plays a moderating role between parental food education and the parent–child relationship, and the moderating effect is greater at the middle school stage than at the elementary school stage. Hypothesis H6: The teaching stage plays a moderating role between motivation and children’s food literacy, with greater moderating effects at the elementary school stage than at the middle school stage.

### 3.2. Participants and Procedure

Five elementary and middle schools in southern China were selected as the survey objects. With input from 62 (30.392%) elementary school students and 142 (69.608%) middle school students, a total of 287 students responded to the questionnaire survey; however, a total of 83 invalid questionnaires were excluded (for reasons such as omitting too many questions, answering the same options, or providing contradictory options), leaving 204 valid questionnaires, and the effective recovery rate was 71.080%. The valid entries were contributed by 98 male students (48.039%) and 105 female students (51.471%), aged 9–14 years (mean age 11.938 years, standard deviation 1.004). This study was approved by the Academic Ethics Committee of the first author’s university (No. GDUTXS2024106). All participants and their guardians provided written informed consent.

The survey was conducted in the form of a paper questionnaire, which was distributed and organized by the participants’ lead teachers, who explained the relevant concepts and behaviors, after which the participants were given ample time to complete the questionnaire according to the given questions, recalling a specific scenario or based on his/her own experience and knowledge, and then immediately return the questionnaire. Before participants filled out the questionnaire, they were first informed of the voluntary and confidential nature of the survey, and that if they felt any discomfort, they could express it to the operator and stop answering the questions. The personal data of the participants involved will be kept strictly confidential, and their data and information will be used for scientific research only. Characteristics of the participants are detailed in Table 1.

### 3.3. Measures

#### 3.3.1. Parental Food Education Scale

A 5-point Likert scale was used to measure the frequency of practical food education (e.g., cooking) and theoretical education (e.g., picture books) used at home by fathers and mothers, respectively, with 4 question items, for which “1” indicated never, and “5” indicated always. The Cronbach’s alpha coefficient for the scale utilized in this study was 0.821.

#### 3.3.2. Parent–Child Relationship Scale

The parent–child relationship scale was adapted from Bong’s (2008) [54] and Park et al.‘s (2004) [55] Children’s Perceived Parent–Child Relationship Scale, Furman and Buhrmester’s (1985) [56] Social Relationship Network Questionnaire, and some of the items from Luo Guoying’s (1997) [57] Perceived Parent–Child Relationship Scale, with a total of 13 items in 3 dimensions, namely, parent–child intimacy, parental message disclosure, and satisfaction with parent–child relationship, with items 6 and 12 using reverse scoring. Higher scores represented a better parent–child relationship, measured independently by fathers and mothers, with 26 question items overall. A 5-point Likert scale was used, in which “1” indicated strong disagreement, and “5” indicated strong agreement. The Cronbach’s alpha coefficient for the scale utilized in this study was 0.901.

#### 3.3.3. Learning Motivation Scale

Adapted from the Learning Motivation Scale used in the study by Hulleman et al. (2010) [58], we studied three dimensions, namely, interest, performance expectation, and utility value. The reliability and validity of this scale were shown to be reliable in subsequent studies [59]. There were 7 question items used in this study, with items 2, 3, and 7 being reverse scored. A 5-point Likert scale was used, where “1” indicated strong disagreement, and “5” indicated strong agreement. The Cronbach’s alpha coefficient for the scale utilized in this study was 0.759.

#### 3.3.4. Children’s Food Literacy Scale

Adapted from scales used in an experiential cooking and nutrition education program by Elizabeth Jarpe-Ratner et al. (2016) [40] and a “Happy Family Kitchen” program by Ho, Cyh et al. (2017) [60], the literacy scale utilized in this study comprised 2 dimensions, namely, children’s dietary quality and their participation in family dietary activities. There were 9 items, with items 2, 4, and 5 being reverse scored. A 5-point Likert scale was used, where “1” indicated never, and “5” indicated always. The Cronbach’s alpha coefficient for the scale utilized in this study was 0.760.

### 3.4. Data Analysis

First, the data were tested for common method bias using the Statistical Package for the Social Sciences (SPSS 27.0), and then descriptive statistics and correlation analyses were used to calculate means, standard deviations, and correlation coefficients for all variables. Finally, PROCESS (Model 6) of SPSS was applied to analyze the mediating roles of the parent–child relationship and learning motivation between parental food education and children’s food literacy, while the moderating roles of the teaching stage between parental food education and parent–child relationship, and of the teaching stage between learning motivation and children’s food literacy, were analyzed using (Model 92), in order to determine whether the relevant paths were moderated by the teaching stage. All variables studied were standardized in Model 6 and Model 92 prior to model testing.

### 3.5. Common Method Bias Test

In this study, Harman’s one-way test was implemented to test for possible common method bias. It was found that 26 factors had initial eigenvalues exceeding 1. In addition, the first common factor accounted for only 26.358% of the overall variance, which was lower than 40.000%. This finding suggests that the data gathered in this study were not influenced by significant common method bias.

## 4. Results

### 4.1. Descriptive Statistics and Correlation Analysis

Pearson’s correlation analysis was used to explore the potential relationships between parental food education, the parent–child relationship, learning motivation, children’s food literacy, and the teaching stage, and the descriptive statistics for each variable are shown in Table 2. In particular, parental food education was significantly and positively correlated with the parent–child relationship, learning motivation, and children’s food literacy; the parent–child relationship was significantly and positively correlated with learning motivation and children’s food literacy; and learning motivation was significantly and positively correlated with children’s food literacy.

### 4.2. Mediating Effect Test

PROCESS Model 6 analysis revealed that parental food education style significantly and positively predicted the parent–child relationship (β = 0.524, *p* < 0.001); parental food education style significantly and positively predicted learning motivation (β = 0.285, *p* < 0.001); and the parent–child relationship was significant and positively predictive of learning motivation (β = 0.259, *p* < 0.001). When parental food education, the parent–child relationship, learning motivation, and children’s food literacy were included in the regression equation at the same time, parental food education was a significant direct predictor of children’s food literacy (β = 0.563, *p* < 0.001), while the parent–child relationship was a non-significant predictor of children’s food literacy (β = −0.045, *p* = 0.508), and learning motivation was a significant direct predictor of children’s food literacy (β = 0.241, *p* < 0.001).

**Figure 2:**
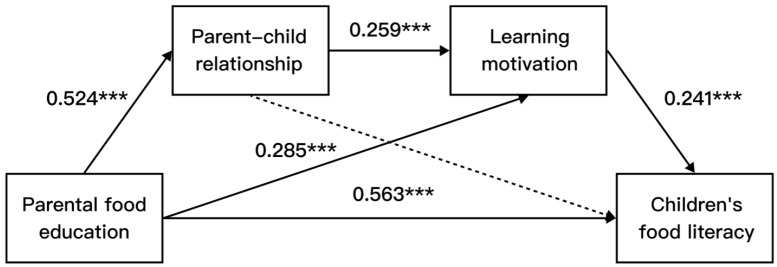
Mediating effect of parental food education on children’s food literacy Note: *** *p* < 0.001.

The analysis of these mediating effects showed (Figure 2 and Table 3) that they consisted of indirect effects generated via two pathways, one of which was indirect effect 2 (0.069), through parental food education → learning motivation → children’s food literacy, and the other being indirect effect 3 (0.033), through parental food education → parent–child relationship → learning motivation → children’s food literacy, while indirect effect 1 (−0.024) was not significant.

In addition, as shown in Table 4 and Table 5, we tested paternal and maternal data separately for mediating effects, and the results indicated that the father’s and mother’s food education had a co-significant educational effect on children’s food literacy, and that the paths of the effects were consistent in their significance.

### 4.3. Moderated Mediation Test

We predicted the teaching stage to produce an association between parental food education and parent–child relationships. PROCESS Model 92 was used to test this hypothesis. The teaching stage moderated the association between parental food education and the parent–child relationship (95% CI [0.023, 0.293], excluding 0) significantly.

**Figure 3:**
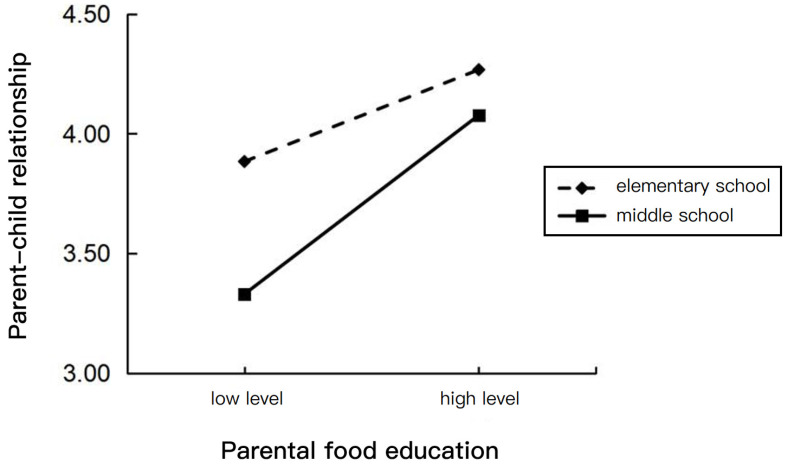
The effect of the interactions of parental food education and the teaching stage on the parent–child relationship.

A simple slope test (Figure 3) showed that, when children were in elementary school, the effect of parental food education on the parent–child relationship was b = 0.166, *p* = 0.004; however, when children were in middle school, the effect of parental food education on the parent–child relationship was b = 0.324, *p* < 0.001. The results indicated that, compared to the elementary school stage of teaching, the middle school stage enhances the effect of parents’ food education on the parent–child relationship.

Conditional indirect effects analyses further indicated that the teaching stage moderated the indirect effect of parental food education on children’s food literacy through the parent–child relationship and learning motivation (95% CI [0.001, 0.023], excluding 0). See Table 6, specifically, which shows that the positive indirect relationship between parental food education and children’s food literacy was significantly weakened when children’s teaching stage was elementary school (indirect effect = 0.010, SE = 0.005, 95% CI = [0.002, 0.021]). The positive indirect relationship between parental food education and children’s food literacy was significantly stronger when children’s teaching stage was middle school (indirect effect = 0.020, SE = 0.008, 95% CI = [0.006, 0.037]).

We predicted the teaching stage to produce an association between learning motivation and children’s food literacy. The PROCESS Model 92 was used to test this hypothesis. The teaching stage moderated the association between learning motivation and children’s food literacy (95% CI [−0.440, −0.029], exclusion 0) significantly.

**Figure 4:**
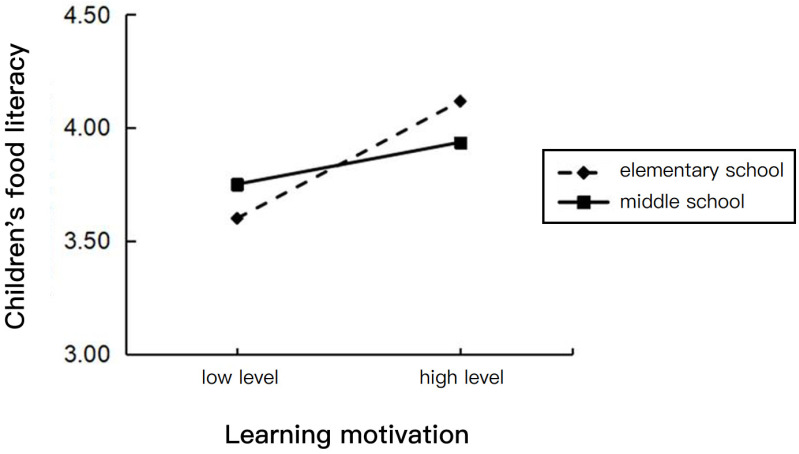
The effect of the interactions of learning motivation and teaching stage on children’s food literacy.

In addition, a simple slope test (Figure 4) indicated that learning motivation had a significant effect on children’s food literacy when children were in the elementary school stage, b = 0.366, *p* < 0.001. However, when children were in the middle school stage, parental food education did not have a significant effect on the parent–child relationship, b = 0.131, *p* = 0.051. The results suggest that, compared to the elementary school teaching stage, the middle school teaching stage attenuated the effect of learning motivation on children’s food literacy.

## 5. Discussion

In this study, we examined the effects of parental food education on children’s food literacy and found that they were mediated through the parent–child relationship and learning motivation, and that this mediating effect arose through two indirect pathways: firstly, through the independent effect of learning motivation; and secondly, through the joint effects of the parent–child relationship and learning motivation. In addition, the effects of parental food education on the parent–child relationship and of learning motivation on children’s food literacy were moderated by the teaching stage. The results of this study can help to explore the mechanism of parental food education on children’s food literacy and provide guiding recommendations for ensuring a healthy family food environment for children in order to prevent chronic diseases, such as obesity.

### 5.1. The Effect of Parental Food Education on Children’s Food Literacy

In the present study, we found that parental food education was significantly and positively associated with children’s food literacy, and that the total effect of parental food education on children’s food literacy was significant and directly predictive. These results confirm that parents are the primary figures in planning diets, making shopping decisions, and determining the extent to which adolescents participate in family food activities [61], and that their food-related education, both theoretical (e.g., picture books) and practical (e.g., cooking), has a significant impact on children’s food literacy in terms of dietary quality and participation in family dietary activities. Research on food education programs suggests that existing food education is characterized by ‘overcooking’, i.e., spending time preparing food to be eaten rather than using food as a learning tool [62], whereas children may be more engaged, competent, and motivated if they are given the time and space to discuss and communicate appropriately [63].

### 5.2. The Mediating Effects of the Parent–Child Relationship and Learning Motivation

In the present study, we found that the parent–child relationship did not play a mediating role between parental food education and children’s food literacy, a result which contradicts our hypothesis. There may be three reasons for this, as follows: Firstly, some studies have come to similar conclusions, that there is no direct association between the quality of the parent–child relationship and the risk of obesity in children; instead, children’s self-regulation has been found to moderate the association between the quality of the parent–child relationship and unhealthy food consumption and higher BMI status [64]. From these findings, it can be hypothesized that there may be an indirect, but not direct, predictive role between the parent–child relationship and children’s food literacy. Secondly, in studies of children’s diets, family members and family structure have been described as the factors that most often influence their daily lives, including parents, siblings, and other adults in the family [61]. A number of surveys have found that the first-born child tends to be given more freedom, that parents feel more responsible for making sure younger children are taken care of than older children, and that older siblings tend to have more control and a sense of familial responsibility over their eating habits and play a greater role in providing food for themselves and others [61]. Thus, family environmental factors such as family members living together, family structure, birth order, and family perceptions all influence children’s food literacy, and the parent–child relationship, as a subsystem of the family dynamic, is not determinative. Thirdly, socio-economic factors have led to family dietary activities becoming a necessary life skill for children, or even a burden [36], as children living in families with lower economic levels are prone to take on the burden of family cooking earlier, making food-related competencies essential for survival, and related food knowledge and skills more abundant, in which case the influence of the parent–child relationship will play a lesser role, and may even lead to a worse parent–child relationship due to the conflict between economic level and food preferences; therefore, children from families with lower economic levels may have a poor parent–child relationship, but will tend to have higher food literacy than children from better-off families because of the demands of life and family responsibilities.

In this study, we found that learning motivation mediated the relationship between parental food education and children’s food literacy, which is consistent with previous research about parenting and involvement increasing children’s learning motivation [22]. Higher learning motivation predicts better learning outcomes, and children’s food literacy is a phenomenal response to the effectiveness of food education. The results of the present study also confirm that the relevant question items containing interest, performance expectations, and utility value, as defined by Hulleman et al. [58], can be used to measure learning motivation in the area of food education. The results suggest that parental food education can be viewed as an education within the family scenario and can be associated with factors related to teaching or learning, which may help to increase the emphasis on food education for both parents and children, as well as enable food educators to pay more attention to the important role of the family food education scenario.

In addition, the results indicate that parental food education increases children’s food literacy, and that this effect is mediated through the combined effects of the parent–child relationship and learning motivation, a finding that not only sheds further light on the mechanism of action by which parental food education affects children’s food literacy, but also confirms that the parent–child relationship can indirectly predict children’s learning outcomes through learning motivation, and that the direct effect is much lower than the indirect effect [65]. Parental food education can increase interactions and communication with children, thus building a good parent–child relationship and, in the process, parental involvement; a close parent–child relationship also allows children to be aware of their parents’ evaluations and expectations of their own food learning, and shapes their self-image in accordance with their parents’ expectations to build up their own personal self-expectations, so that children’s learning motivation is enhanced, thus shaping better food literacy through better learning behaviors.

### 5.3. The Moderating Effect of the Teaching Stage

In this study, according to the available data, we found that the teaching stage significantly moderated the effects of parental food education on the parent–child relationship and of learning motivation on children’s food literacy. However, due to the small amount of data in the two groups of the teaching stage, it is not rigorous and scientific, so the discussion on the role of the teaching stage in this study is only to expand and supplement it. Firstly, the positive effect of parents’ food education on the parent–child relationship was increased for middle school students compared to elementary school students; secondly, the positive effect of learning motivation on elementary school students’ food literacy was increased for them compared to for middle school students. The above findings are consistent with the results of the previous hypotheses. On the one hand, as middle school students spend less time with their parents due to the change in teaching stage, there is a more complex social network to which to adapt, affecting children’s self-perception and overall self-esteem [23] and making the parent–child relationship more vulnerable, so when the frequency of parental food education decreases, middle school students have even fewer opportunities to have a strong relationship with their parents, which leads to poorer relationship quality. On the other hand, the results of this study showed that middle school students scored significantly higher on nutrition knowledge than elementary school students, but elementary school students scored significantly higher on nutrition practices [52]; however, higher levels of knowledge do not necessarily lead to more favorable nutritional practices and behaviors [66]. Elementary school students’ food intake reflects their parents’ choices and functions more than their own knowledge-based behaviors, whereas middle school students make more independent food choices [52], which shows that, from elementary school to middle school, children gain more freedom and a sense of autonomy in the area of food, and are richer in related knowledge, but a more complex food environment may cause middle school students to shift their food literacy, which was cultivated by family dominance in the elementary school stage, and to become more independent in their thoughts and judgments; thus, food is no longer as simple as education, but may be a form of socialization, or even identity, so that the food literacy of middle school students is less affected by the learning motivation for food education.

### 5.4. Research Suggestions and Limitations

This study is of great significance for enabling parents to conduct food education in the family, create a favorable family food environment, improve children’s food literacy in early adolescence, promote their physical and mental health development, and prevent chronic diseases, such as obesity. On the one hand, regardless of the school or family food program, attention should be paid to the specific form of parents’ approach to food education, increasing parent–child communication and interaction, and increasing children’s motivation to learn food-related knowledge through parental encouragement and care, so as to improve their food literacy. On the other hand, we found that middle school students demonstrated a more significant moderating effect from parental food education on the parent–child relationship, and elementary school students showed a more significant moderating effect from learning motivation on their food literacy, which suggests that, when planning or designing a family food education model, increasing parental involvement in food education can significantly improve the parent–child relationship for middle school students, while increasing children’s learning motivation can significantly improve elementary school children’s food literacy, and the relevant elements can be adjusted to improve children’s food literacy through the different teaching stages.

In addition, some limitations of this study need to be addressed. Firstly, in the present study, we measured data related to fathers and mothers at the same time, and it was difficult to distinguish the individual influences of fathers or mothers on children’s food in the same family setting, so we were unable to analyze whether there were differences in the influences of individual parents on children’s food literacy. Secondly, the present study was based entirely on children’s self-reports, which could be seen as a potential strength, as children’s perceptions of the parent–child relationship may have the greatest influence on their behavior, but children’s subjective experiences of the parent–child relationship do not automatically correspond to observable data [67]; thus, measures of the parent–child relationship may suffer from cognitive bias. Thirdly, in this study, we only measured and analyzed the parent–child relationship, lacking attention to the influences of other family members or the family environment; therefore, it is recommended that future research should consider a broader range of family contexts, including all family members’ relationship systems, the family’s economic situation, parents’ education level, and familial perceptions, among others. Finally, there is a large difference between the samples of elementary and middle school students surveyed in this study, with the sample of middle school students being larger than that of elementary school students; it is recommended that future research analyze the proposed model by considering equal approximations of the two sample sizes for analysis of variance (ANOVA).

## 6. Conclusions

The results of the present study showed that parental food education positively predicted parent–child relationship, learning motivation, and children’s food literacy; in food education, the parent–child relationship positively predicted learning motivation, but did not directly predict children’s food literacy; meanwhile, learning motivation positively predicted children’s food literacy, which implies that parental involvement in children’s food education increases the parent–child relationship, enhances children’s learning motivation, and shapes children’s increased food literacy. Specifically, parental food education influences children’s food literacy by affecting learning motivation; it also influences children’s food literacy sequentially, through the parent–child relationship and learning motivation.

## Figures and Tables

**Table 1:** Demographics characteristics (n = 204).

Category		Frequency
	≤10	24 (11.8%)
	11	30 (14.8%)
	12	84 (41.1%)
	≥13	65 (32.0%)
Gender	Male	98 (48.3%)
	Female	105 (51.7%)
Teaching Stage	Elementary School Stage	62 (30.4%)
	Middle School Stage	142 (69.3%)
Only Child	Yes	37 (18.1%)
	No	167 (81.9%)

**Table 2:** Mean, standard deviation, and correlation matrix for each variable.

	M	SD	1	2	3	4	5
1. Teaching stage	-	-	-				
2. Parental food education	2.827	1.153	−0.116	-			
3. Parent–child relationship	3.807	0.640	−0.312 **	0.524 **	-		
4. Learning motivation	3.657	0.708	−0.002	0.421 **	0.408 **	-	
5. Children’s food literacy	3.849	0.618	−0.056	0.563 **	0.307 **	0.427 **	-

Note: ** Significant correlation at 0.01 level (two-tailed). M: mean. SD: standard deviation.

**Table 3:** Bootstrap 95% confidence intervals for mediating effect pathways.

	Effect	Boot SE	Boot LLCI	Boot ULCI	Relative Mediation Effect
Ind1: Parental food education → parent–child relationship → children’s food literacy	−0.024	0.038	−0.100	0.050	-
Ind2: Parental food education → learning motivation → children’s food literacy	0.069	0.030	0.019	0.133	22.85%
Ind3: Parental food education → parent–child relationship → learning motivation → children’s food literacy	0.033	0.013	0.010	0.062	10.93%

Note: Boot SE: Bootstrap standard error. Boot LLCI: Bootstrap lower limit confidence interval. Boot ULCI: Bootstrap upper limit confidence interval. Ind: Indirect effect.

**Table 4:** Bootstrap 95% confidence intervals for father-mediated effect pathways.

	Effect	Boot SE	Boot LLCI	Boot ULCI	Relative Mediation Effect
Ind1: Father’s food education → parent–child relationship → children’s food literacy	−0.044	0.040	−0.124	0.031	-
Ind2: Father’s food education → learning motivation → children’s food literacy	0.078	0.030	0.027	0.145	30.56%
Ind3: Father’s food education → parent–child relationship → learning motivation → children’s food literacy	0.028	0.014	0.003	0.059	10.94%

Note: Boot SE: Bootstrap standard error. Boot LLCI: Bootstrap lower limit confidence interval. Boot ULCI: Bootstrap upper limit confidence interval. Ind: Indirect effect.

**Table 5:** Bootstrap 95% confidence intervals for mother-mediated effect pathways.

	Effect	Boot SE	Boot LLCI	Boot ULCI	Relative Mediation Effect
Ind1: Mother’s food education → parent–child relationship → children’s food literacy	0.010	0.033	−0.055	0.074	-
Ind2: Mother’s food education → learning motivation → children’s food literacy	0.070	0.030	0.019	0.135	25.45%
Ind3: Mother’s food education → parent–child relationship → learning motivation → children’s food literacy	0.029	0.012	0.008	0.054	10.46%

Note: Boot SE: Bootstrap standard error. Boot LLCI: Bootstrap lower limit confidence interval. Boot ULCI: Bootstrap upper limit confidence interval. Ind: Indirect effect.

**Table 6:** Bootstrap results of teaching stage.

Teaching Stage	Boot Indirect Effect	Boot SE	95% CI	
			Lower	Upper
elementary school	0.010	0.005	0.002	0.021
middle school	0.020	0.008	0.006	0.037

Note: Number of Bootstrap samples: 5000. SE: standard error. CI: confidence intervals.

## Data Availability

The data presented in this study are available on request from the corresponding author.
